# MicroRNA expression and oxidative stress markers in pectoral muscle of broiler chickens fed diets supplemented with phytobiotics composition

**DOI:** 10.1038/s41598-024-54915-y

**Published:** 2024-02-22

**Authors:** Karolina A. Chodkowska, Marcin Barszcz, Anna Tuśnio

**Affiliations:** 1 Krzyżanowski Partners Spółka z o.o., Zakładowa 7, 26-670 Pionki, Poland; 2 AdiFeed Sp. z o.o., Opaczewska 43, 02-201 Warszawa, Poland; 3 Department of Animal Nutrition, The Kielanowski Institute of Animal Physiology and Nutrition, Polish Academy of Sciences, Instytucka 3, 05-110 Jabłonna, Poland

**Keywords:** miRNA, Target genes, Gene ontology, Muscle, Lipid peroxidation, Phytobiotics, Broiler, Myoprotection, Production parameters, Genetics, Molecular biology, Physiology, Plant sciences, Zoology

## Abstract

Phytobiotic compositions are commercially used in broiler production, mostly to improve general health and the production parameters. Moreover, some of their active substances may change the expression of miRNA in different tissues. Therefore, the purpose of this study was to evaluate the effect of the phytobiotic composition (PBC) containing white mustard, calamus, turmeric, and common ivy on production parameters, oxidative stress markers and expression of selected miRNAs in pectoral muscle of broiler chickens. The experiment was performed on broiler chickens fed the control diet (without PBC), and a diet supplemented with 60 or 100 mg/kg of PBC for 35 days. After the experiment, samples (blood and muscle) were collected for analyses. The analyzed production parameters included: feed conversion ratio, feed intake and body weight. There was no effect on growth performance of broiler chickens but feeding diet supplemented with 60 mg/kg phytobiotics significantly increased the expression of miR-30a-5p, miR-181a-5p, and miR-206, and decreased that of miR-99a-5p, miR-133a-5p, miR-142-5p, and miR-222 in pectoral muscle of chickens. The addition of 100 mg/kg phytobiotics significantly increased miR-99a-5p and miR-181a-5p expression, and caused down-regulation of the expression of miR-26a-5p and miR-30a-5p. Chickens fed diet supplemented with 100 mg/kg PBC had lower level of lipid peroxidation products in blood, while in the muscle tissue it was higher in birds fed a diet with the addition of 60 mg/kg as compared to the control group. The results suggest that this unique composition of phytobiotics does not affect productive traits but can change expression of miRNAs that are crucial for muscle physiology and pathology in broiler chickens. This additive may also protect against the oxidative stress but the effect is dose dependent.

## Introduction

Phytobiotics are commercially used feed additives in broiler production around all over the world. Previous observation showed that dietary addition of white mustard (*Sinapis alba* L.), calamus (*Acorus calamus* L.), turmeric (*Curcuma longa* L.), and common ivy (*Hedera helix* L.) mixture may be an effective alternative for coccidiostats, particularly in a production according to antibiotic reduction programmes^1^. The effects of this phytobiotic composition on broilers may be broader and comparable to antibiotic growth promoters. White mustard is a source of a well-known glucosinolate, sinalbin, which is mostly recognised as an antimicrobial agent but also showed an antioxidant, proapoptotic, and antiproliferative effect (related to the modulation of the MAPK pathway)^2^. Also, it was shown that white mustard enhanced the activity of several antioxidant enzymes, such as superoxide dismutase, catalase, and glutathione peroxidase^3^. Calamus, similarly to white mustard, presented antimicrobial and antioxidant activity^4–6^. Moreover, number of studies showed that it has anti-inflammatory and wound-healing properties^7,8^. In poultry, several studies related to calamus were performed. Most of them were associated with coccidiosis prevention and control^9^, antioxidant activity and cholesterol concentration of quails’ eggs^10^. Turmeric has a several properties that were investigated in many studies related to different tissues and animal models. In broiler chickens the effect of turmeric addition on growth performance and meat quality was also analysed^11^. Moreover, it was found that dietary supplementation with turmeric alleviated the aflatoxin-induced down-regulation of *sod*, *gst*, *eh* gene expressions, and up-regulation of *il-6*, *cyp1a1*, and *cyp2h1* in the liver of chickens^12^. These results demonstrated beneficial effects of turmeric on antioxidant defence, biotransformation, and immune system of the liver of chicks challenged with aflatoxin B. Feeding diet supplemented with turmeric may also improve antioxidant defence in blood^13^. Common ivy is another constituent of phytobiotic composition with saponins being its most important bioactive compounds. Saponins may affect growth performance of broiler chickens, meat quality, and can be used for the control of coccidiosis and other diseases such as necrotic enteritis or colibacillosis^14–16^.

Only a few studies have been performed on the effect of phytobiotic composition on miRNA expression so far. Most of them were related to mammal, human and cell culture models and only singe studies were performed on a broiler chickens^17–19^. MicroRNAs (miRNAs) are small, non-coding, interfering molecules of RNA that regulate gene expression through the sequence-specific base pairing to messenger RNA (mRNA). Previous studies showed that miRNAs are related to the key physiological and pathological processes in various tissues in animals and may also be a great diagnostic tool also in veterinary medicine or animal production^20^. Moreover, in mammal models, there is a group of miRNAs, called myomirs, that are highly enriched in skeletal and/or cardiac muscles and includes: miR-1, miR-133a, miR-133b, miR-206, miR-208, miR-208b, miR-486. These miRNAs are involved in skeletal muscle development. Previous studies showed that the expression of these miRNAs may be modulated by several natural nutriceutical substances^21,22^. In birds, there are only a few studies related to myomirs that may be associated with physiological and pathological processes in muscle tissue.

The oxidative stress is a phenomenon that may contribute to the development of skeletal muscle pathogenesis but its role is extremely complex. Transient oxidative stress may be beneficial, on the other hand, uncontrolled production and accumulation of reactive oxygen species might have pathological implications^23^. It is well known that oxidative stress may induce muscle aging and myopathies like white stripping or wooden breast^24,25^. Moreover, it has been demonstrated that dietary antioxidants could combat oxidative stress and improve broiler performance as well as meat quality^26^. However, it is unknown how natural antioxidants present in phytobiotic composition (PBC) affect oxidative stress markers in muscle tissue and whether they influence the expression of miRNA related to muscle development. The research hypothesis assumed that bioactive compounds present in the phytobiotic mixture will beneficially affect muscle tissue. Therefore, the aim of the study was to determine the effect of dietary level of PBC containing white mustard, calamus, turmeric, and common ivy on growth performance, miRNA expression in pectoral muscle and oxidative stress markers in broiler chickens.

## Results

### Production parameters

In production parameters being recorded, i.e. body weight (BW), feed intake (FI) and feed conversion ratio (FCR), no differences between groups were observed (Table 1). The highest body weight was observed in a group fed with 60 mg/kg of phytobiotics mixture. Moreover, also this group presented the lowest FCR.

**Table 1:** Growth performance parameters of broiler chickens fed diet supplemented with phytobiotic addition.

Phytobiotic addition (mg/kg)	Body weight (g)	Feed intake (g)	Feed conversion ratio
0	2388	3072	1.29
60	2452	3037	1.24
100	2361	2943	1.25
SEM	23	38	0.01
P-value	0.265	0.369	0.185

### MicroRNA expression

Feeding diet supplemented with 60 mg/kg phytobiotics significantly increased the expression of miR-30a-5p, miR-181a-5p, and miR-206 and decreased that of miR-99a-5p, miR-133a-5p, miR-142-5p, and miR-222 in pectoral muscle of chickens. The expression of miR-26a-5p was unaffected by the treatment (Table 2).

**Table 2:** The effect of dietary supplementation with 60 mg/kg phytobiotic composition on miRNA expression in pectoral muscle of broiler chickens in comparison with the control group (expression = 1).

No	miRNA	Expression	SEM	P-value	Regulation
1	miR-26a-5p	1.32	2.328	0.095	No difference
2	miR-30a-5p	1.53	2.523	0.003	Up
3	miR-99a-5p	0.21	0.387	< 0.001	Down
4	miR-133a-5p	0.16	0.289	< 0.001	Down
5	miR-142-5p	0.17	0.316	< 0.001	Down
6	miR-181a-5p	1.46	0.574	0.045	Up
7	miR-206	3.60	6.683	0.042	Up
8	miR-222	0.18	0.340	< 0.001	Down

In pectoral muscle of chickens fed a diet with the addition of 100 mg/kg phytobiotics, there was a significant increase of miR-99a-5p and miR-18a-5p expression. Feeding this diet caused significant down-regulation of the expression of miR-26a-5p and miR-30a-5p and had no effect on miR-133a-5p, miR-142-5p, miR-206, and miR-222 expression (Table 3).

**Table 3:** The effect of dietary supplementation with 100 mg/kg phytobiotic composition on miRNA expression in pectoral muscle of broiler chickens in comparison with the control group (expression = 1).

No	miRNA	Expression	SEM	P-value	Regulation
1	miR-26a-5p	0.45	0.832	< 0.001	Down
2	miR-30a-5p	0.51	0.929	< 0.001	Down
3	miR-99a-5p	1.35	0.382	0.025	Up
4	miR-133a-5p	1.25	0.296	0.462	No difference
5	miR-142-5p	1.57	0.399	0.052	No difference
6	miR-181a-5p	1.36	0.279	0.047	Up
7	miR-206	0.75	1.417	0.567	No difference
8	miR-222	1.37	0.360	0.064	No difference

#### Functional analysis of identified miRNAs

**Figure 1:**
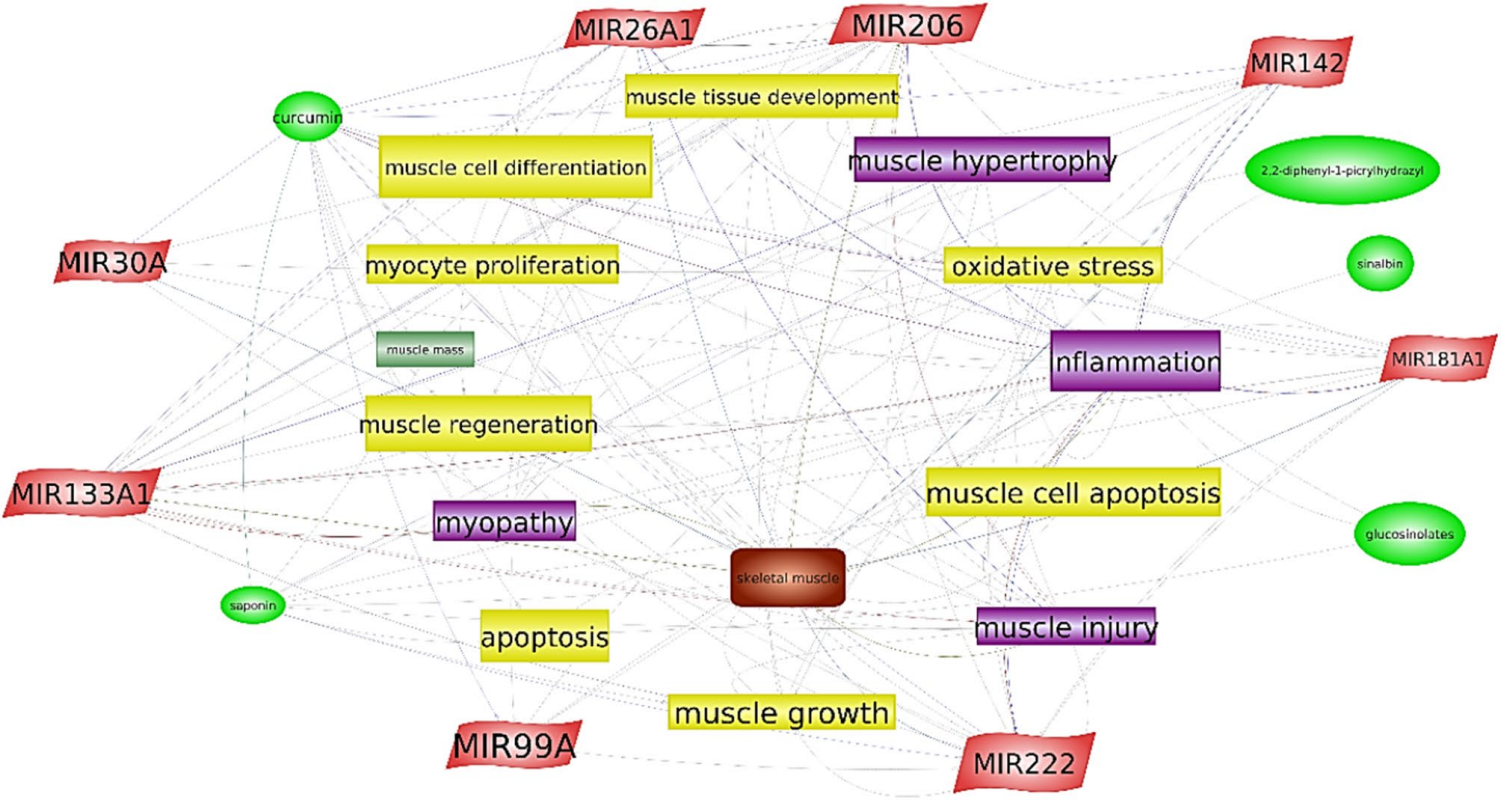
Links between selected miRNAs, single components of phytobiotics mixture and their involvement in some selected physiological and pathological processes in a muscle tissue.

Based on the Pathway Studio Mammal Plus Web Software 12.5 (Elsevier, USA) analysis and available literature a number of links between the individual components of the phytobiotic mixture, key physiological and pathological processes in muscle tissue and the miRNAs analyzed were found (Fig. 1). Selected relations in details are presented in Pathway Studio Summary List as single figures and tables (Supplementary material 1, 1a). Interestingly, this type of analysis has shown that the links of the individual components of the product to specific processes are already known, but also their relationships with miRNAs related to these processes. Among the key processes that have been described earlier in works on the impact of phytobiotics are: muscle cell differentiation and proliferation, muscle hypertrophy, muscle tissue development processes, muscle damage and repair processes, as well as apoptosis and oxidative stress. The abovementioned processes were also described in muscle tissue as those associated with the analyzed miRNA:

- muscle tissue development, muscle cell proliferation and differentiation: miR-26a, miR-30a, miR-99a, miR-133a, miR-206, miR-222, miR-181a,
- muscle hypertrophy, muscle growth: miR-133a, miR-142, miR-181, miR-142,
- muscle regeneration: miR-133a, miR-142,
- inflammation: miR-26a, miR-206, miR-142,
- cell apoptosis: miR-181a, miR-142, miR-133, miR-222, miR-206,
- muscle injury and myopathy: miR-99, miR-181a,
- oxidative stress: miR-142, miR-222.

#### Prediction and ontological analysis of miRNA target genes (TG)

In order to identify dedicated target genes, set of eight statistically significant miRNAs was analysed using TargetScan Release 8.0 (David Bartel and Whitehead Institute Bioinformatics and Research Computing Group, USA). Analysis showed 4845 unique targets for all miRNAs (Supplementary Material; Table S2). After removing duplicated genes, 3216 unique targets were identified. Among them, only 2953 genes were previously described in *Gallus gallus*. Among the group of eight miRNAs, the highest regulation potential to the target genes was presented by three miRNAs: miR-222, miR-142-5p; (936 identified target genes for each of them), and miR-133 (816 identified target genes). The lowest regulation potential was noticed for miR-99a-5p, i.e. 30 identified target genes.

#### Ontological analysis of all identified potential target genes

Functional analysis using Pathway Studio, D.A.V.I.D. 6.7 and Panther 17.0 software showed that all TG for analysed miRNAs were associated with several processes which may be key players in several physiological (protein metabolism, muscle tissue development, apoptosis) and pathological (inflammation, oxidative stress, myopathies, myositis) conditions in muscle tissue (Supplementary Material; Table S3). Moreover, functional network analyses of target genes of differently expressed miRNAs (based on KEGG data^27^) showed their involvement in regulation of actin cytoskeleton, MAPK signalling, FoxO signalling pathway, Wnt signalling pathway, TGF-β signalling pathway, ubiquitin mediated proteolysis, and mTOR signalling pathway suggesting their involvement in breast muscle growth (also on protein metabolism level) in chickens (Table 4).

**Table 4:** Selected pathways for all identified target genes (based on the D.A.V.I.D. analysis). Several of them (MAPK signaling pathway, FoxO, Wnt etc.) were previously described as crucial pathways in muscle tissue physiology and pathology.

Category	Term	Count	%	P-value	Benjamini	FDR	Fisher Exact
KEGG_PATHWAY	Focal adhesion	76	2.6	8.50E−11	1.20E−08	1.00E−08	3.10E−11
KEGG_PATHWAY	Regulation of actin cytoskeleton	73	2.5	2.30E−10	1.70E−08	1.40E−08	8.50E−11
KEGG_PATHWAY	MAPK signaling pathway	81	2.7	1.30E−09	6.20E−08	5.20E−08	5.30E−10
KEGG_PATHWAY	Adherens junction	33	1.1	5.90E−07	1.70E−05	1.40E−05	1.60E−07
KEGG_PATHWAY	FoxO signaling pathway	49	1.7	4.80E−07	1.70E−05	1.40E−05	1.80E−07
KEGG_PATHWAY	Wnt signaling pathway	48	1.6	9.80E−07	2.40E−05	2.00E−05	3.70E−07
KEGG_PATHWAY	ErbB signaling pathway	35	1.2	1.70E−06	3.40E−05	2.90E−05	5.10E−07
KEGG_PATHWAY	mTOR signaling pathway	25	0.8	6.50E−06	1.00E−04	8.80E−05	1.60E−06
KEGG_PATHWAY	TGF-beta signaling pathway	33	1.1	5.80E−06	1.00E−04	8.80E−05	1.90E−06
KEGG_PATHWAY	Insulin signaling pathway	44	1.5	1.50E−05	2.20E−04	1.80E−04	6.10E−06
KEGG_PATHWAY	Dorso-ventral axis formation	14	0.5	7.60E−05	1.00E−03	8.40E−04	1.20E−05
KEGG_PATHWAY	Oocyte meiosis	34	1.2	1.80E−04	2.10E−03	1.80E−03	7.20E−05
KEGG_PATHWAY	Ubiquitin mediated proteolysis	43	1.5	2.10E−04	2.30E−03	2.00E−03	9.90E−05
KEGG_PATHWAY	Adrenergic signaling in cardiomyocytes	39	1.3	4.80E−04	5.00E−03	4.20E−03	2.30E−04
KEGG_PATHWAY	Insulin resistance	34	1.2	8.60E−04	8.30E−03	7.00E−03	4.00E−04
KEGG_PATHWAY	Phosphatidylinositol signaling system	32	1.1	1.40E−03	1.30E−02	1.10E−02	6.70E−04
KEGG_PATHWAY	Hedgehog signaling pathway	11	0.4	4.60E−03	3.30E−02	2.80E−02	1.10E−03
KEGG_PATHWAY	Gap junction	28	0.9	2.70E−03	2.20E−02	1.80E−02	1.20E−03
KEGG_PATHWAY	Endocytosis	63	2.1	2.50E−03	2.10E−02	1.80E−02	1.50E−03
KEGG_PATHWAY	Melanogenesis	30	1	4.30E−03	3.30E−02	2.80E−02	2.10E−03
KEGG_PATHWAY	Progesterone-mediated oocyte maturation	26	0.9	5.60E−03	3.90E−02	3.20E−02	2.60E−03
KEGG_PATHWAY	Protein processing in endoplasmic reticulum	43	1.5	8.70E−03	5.80E−02	4.80E−02	5.20E−03
KEGG_PATHWAY	Regulation of autophagy	10	0.3	2.20E−02	1.20E−01	1.00E−01	7.00E−03
KEGG_PATHWAY	RNA degradation	22	0.7	1.50E−02	9.30E−02	7.80E−02	7.50E−03
KEGG_PATHWAY	ECM-receptor interaction	24	0.8	1.60E−02	9.30E−02	7.80E−02	8.00E−03
KEGG_PATHWAY	GnRH signaling pathway	25	0.8	1.60E−02	9.30E−02	7.80E−02	8.20E−03
KEGG_PATHWAY	Calcium signaling pathway	45	1.5	1.90E−02	1.10E−01	8.80E−02	1.20E−02
KEGG_PATHWAY	VEGF signaling pathway	19	0.6	3.00E−02	1.60E−01	1.30E−01	1.50E−02
KEGG_PATHWAY	Salmonella infection	21	0.7	3.50E−02	1.80E−01	1.50E−01	1.90E−02
KEGG_PATHWAY	Notch signaling pathway	15	0.5	4.90E−02	2.30E−01	1.90E−01	2.30E−02
KEGG_PATHWAY	Glycosaminoglycan biosynthesis—chondroitin sulfate/dermatan sulfate	8	0.3	7.30E−02	3.20E−01	2.70E−01	2.60E−02
KEGG_PATHWAY	Inositol phosphate metabolism	21	0.7	4.80E−02	2.30E−01	1.90E−01	2.60E−02
KEGG_PATHWAY	mRNA surveillance pathway	21	0.7	5.50E−02	2.50E−01	2.10E−01	3.10E−02

### Oxidative stress markers

Dietary level of phytobiotic did not affect prooxidant-antioxidant balance in blood and pectoral muscle of chickens but there was an effect on thiobarbituric acid-reactive substances (TBARS) concentration (Table 5). Chickens fed diet supplemented with 100 mg/kg PBC had lower TBARS level in blood, while in the muscle tissue it was higher in birds fed diet with the addition of 60 mg/kg as compared with the control group.

**Table 5:** Oxidative stress markers in blood plasma and pectoral muscle of broiler chickens fed diets supplemented with phytobiotic addition.

Phytobiotic addition (mg/kg)	PAB (HKU/g)		TBARS (nmol/g)	
	Blood	Muscle	Blood	Muscle
0	955	7434	0.32^b^	1.63^a^
60	1069	7469	0.25^ab^	4.59^b^
100	1219	7086	0.24^a^	2.26^ab^
SEM	63.9	205.9	0.014	0.456
P-value	0.246	0.718	0.039	0.012

*PAB* prooxidant-antioxidant balance, *TBARS* thiobarbituric acid-reactive substances.

^a,b^Means within columns with different superscript letters differ significantly.

## Discussion

There was no effect on analyzed production parameters (BW, FI, FCR). Despite the lack of statistically significant differences between the control group and both groups where the supplement was administered, it should be emphasized that the best (lowest) FCR and the highest final BW were obtained in the group that received the supplement at a dose of 60 mg/kg. The lack of significant differences may be due to several factors. One of them may be too low doses of a phytobiotic additive, which is in line with previous observations on another phytobiotic supplement with similar composition^19^. The second factor that may affect the obtained results is the duration of the experiment, i.e., 35 days, which may be too short period to observe an improvement in production parameters. However, it is sufficient to improve the parameters related to oxidative stress or to change the expression of selected miRNAs involved in physiological and pathological processes in muscle tissue.

Analysis of the expression of selected miRNAs showed differences in both experimental groups as compared to the control group. Interestingly, only one miRNA (miR-181a-5p) showed consistent direction of expression for both groups. The remaining miRNAs showed different expression directions for each group, or their expression level was negligible for either group. This clearly indicates a dose-dependent phenomenon and it is necessary to carry out more detailed analyses for each of the tested doses. Moreover, the results suggest that a 60 mg/kg dose seems to positively affect miRNAs that are involved in processes of muscle growth and muscle protection. On the other hand, the results of miRNAs for a higher dosage may indicate that 100 mg/kg may have no effect on muscle growth and myoprotection.

Most of analysed miRNAs are known as myomiRs or are closely related to physiology and pathology of muscle tissue (miR-133, miR-206, miR-222, miR-142, miR-99a-5p and miR-181a-5p). The crucial roles of two muscle-specific miRNAs, i.e., miR-133 and miR-206, in the regulation of myogenesis have been well documented^28,29^ in human or other mammal models but only single papers were associated with birds. What is interesting, the down-regulation of miR-133a-5p, miR-222, miR-99a-5p, and miR-181a-5p and up-regulation of miR-206 and miR-30a-5p in pectoral muscle of chickens fed a diet supplemented with 60 mg/kg phytobiotic composition is in line with the direction of changes observed for the intensification of muscle cell differentiation (also in birds) and the growth of muscle tissue^30–32^. One miRNA, miR-142, seems to be also interesting in the hypertrophy aspect. Its downregulation was described during cardiac hypertrophy and is able to inhibit cytokine signalling and function in the myocardium^33^. Moreover, the same direction of expression was noticed previously after using β-hydroxy-β-methylbutyrate, which is known as a muscle mass builder, in equine satellite cells^22^. As it was mentioned above, only one miRNA, i.e., miR-181a-5p had the same direction of expression in both groups fed phytobiotic-supplemented diets. Surprisingly, in previous studies on human and other mammals up-regulation of its expression was observed in muscle tissue/cells that were during the aging process and inhibited regeneration/proliferation^34^. However, these studies also indicated that overexpression of miR-181a-5p facilitated the differentiation of muscle cells.

Surprisingly, in group of chickens fed diet with higher level of phytobiotics (100 mg/kg) the expression of only four miRNAs was significantly altered. These miRNAs (miR-26a-5p, miR-30a-5p, miR-99a-5p, and miR-181a-5p) were previously described as those involved in muscle cell proliferation/differentiation, muscle aging, muscle atrophy and regeneration^35^. Among the analysed miRNAs, miR-99a seems to be the most interesting, as the latest research indicated that it is involved in the processes of proliferation and differentiation in chicken breast muscles^36^. Authors noticed that knock down of this miRNA may decrease proliferation of muscle cells while its overexpression promoted the cell viability. These results suggest that different doses of phytobiotic mixture may differently affect the same miRNAs and associated processes.

While the majority of the analysed miRNAs were previously described as those associated mainly with the physiological and pathological processes of muscle tissue, and single ones with oxidative stress, apoptosis, the phenomenon of tissue regeneration, etc., the target genes show that a large group of them is also clearly related to the characteristic processes for muscle tissue. Pathway analysis for TG clearly shows that large groups of genes are associated with key metabolic pathways in muscle tissue, i.e., MAPK pathways involved in protein degradation in muscle cells^37^, and oxidative stress^38^. Moreover, MAPK pathway is a key player in a muscle energy metabolism through modulating lipid metabolism, skeletal muscle growth, and also involved with muscular myopathies and atrophy^37,39^. Among identified pathways also FOXO signalling is closely related to muscular dystrophies/atrophy and possibility to modulate the expression of related genes seems to be an effective tool in controlling this kind of muscle disorders^40^. In addition to the pathways mentioned, Wnt signalling, which plays an essential role during embryonic muscle development and is responsible for skeletal muscle homeostasis in the adult^41^, mTOR, which controls both the anabolic and catabolic signalling of skeletal muscle mass and finally modulate the muscle hypertrophy and muscle wastage, deserves attention^42^. The analysis of signalling pathways in terms of the phytobiotic mixture used is interesting because there are single studies on selected ingredients of the additive tested in the current study, confirming the effect on some of them, e.g., curcumin and mTOR signalling pathway^43^ or common ivy and the MAPK pathway^44^. It should be emphasized, however, that the presented analyses are purely theoretical and the obtained results should be verified using dedicated methods based, among others, on gene silencing in specific cell or animal models.

Due to previous reports on the potential antioxidant effect of individual components of the phytobiotic mixture, the miRNA related to this process was selected for the analysis and two oxidative stress markers, TBARS and PAB, were assessed. The method of PAB analysis was established to be used for the evaluation of age-related and metabolic diseases such as diabetes in humans and can be useful in monitoring antioxidant therapy effectiveness^45^. Since the method was designed to evaluate the balance in biological samples such as blood and urine^45^, an attempt was made to applied it in animal studies to evaluate antioxidant effect of chicory inulin in young pigs^46,47^. The PAB was successfully determined in blood plasma as well as in tissue homogenates. Its analysis revealed antioxidant effect of 2% and 3% dietary addition of inulin and 4% dietary level of dried chicory root in blood plasma and liver of 50-day old pigs^46,47^. Lower PAB values indicated the prevalence of antioxidants in a sample and improved antioxidant status. Thus, it serves as a useful marker in studies related to oxidative stress prevention. To the best of our knowledge, this is the second study in which PAB was determined in chicken blood and muscle tissue. The first one, published also by our research group, demonstrated very high levels of balance values in broiler chickens fed diets supplemented with different doses of PBC consisting of red pepper fruit, white mustard seeds, turmeric, soapwort root, and calamus rhizome^48^. Our analyses revealed that the balance values in broiler chicken blood were even ten times greater than those previously determined in pigs^46,47^. Also, PAB values measured in pectoral muscle of chickens were considerably greater than those measured in the liver and kidney of pigs^47^. However, this did not have to mean that there was a prevalence of oxidants in broiler chicken body. The results might rather reflect species specificity and suggested a necessity to adjust the scale of PAB values for chickens. In the current study, blood plasma was diluted ten times prior to the analysis to fit the linear range of the assay. After dilution, the raw absorbance values (0.4–0.8) were in the bottom range of the standard curve and indicated low level of oxidants and high content of antioxidants in blood (35–138 HKU). Similarly, the raw results obtained for muscle supernatants were in the range of 40–70 HKU and also indicated the prevalence of antioxidants in pectoral muscle of broiler chickens. Nonetheless, despite the antioxidative activity of constituents of the phytobiotic composition used in the current study, there was no improvement in the PAB values in blood and muscle tissue, which is in line with our previous research^48^. Probably, the level of dietary supplementation was too low to increase the concentrations of antioxidants circulating in the blood and their accumulation in peripheral tissues. Another reason might be the lack of phytobiotic effect on blood level of small-molecule antioxidants such as uric acid, creatinine, and bilirubin. Unfortunately, these parameters were not measured in the current study, therefore further research is required. Despite the lack of effect on PAB values, feeding diets supplemented with the phytobiotic composition reduced the level of lipid peroxidation products in the blood of chickens. On the other hand, dietary inclusion of 60 mg/kg phytobiotics increased the concentration of TBARS in pectoral muscle of broiler chickens in comparison with the other groups. This is difficult to explain, especially as the miR-142-5p expression in pectoral muscle was down-regulated in this group. As suggested previously^49^, inhibition of miR-142-5p in the liver cell line might upregulate the protein level of nuclear factor-erythroid 2-related factor 2 (Nrf2) and its target antioxidant genes (*ho1*, *nqo1*, *mn-sod*), and in consequence, reduce the production of reactive oxygen species and oxidative stress. However, the role of miR-142-5p in oxidative stress in muscle tissue of broiler chickens may differ from its role in the liver cell line. It was suggested that tissue or cell line specificity could be responsible for discrepancies in miR-142-5p expression during aging process in different tissues and pointed out the need of care in the selection of this miRNA as a marker for detection of tissue or organ aging^50^. It may be also truth in the case of its use as a marker of oxidative stress in muscle tissue. Nonetheless, the current study suggests that the antioxidant effect of the phytobiotic composition is dose-dependent and tissue-specific. Similar results were obtained on pigs^47^, in which antioxidant effect of chicory inulin was found in the blood and liver but not in the kidneys.

## Materials and methods

### Birds, housing, and experimental design

The trial involved 48 one-day-old female Ross 308 broiler chickens, purchased from a local commercial hatchery. Birds were divided into three groups (n = 16) fed cereal-based diets (starter, grower, and finisher) without the addition of PBC (control) or supplemented with 60 or 100 mg/kg of PBC (AdiMax^®^ AP, AdiFeed Sp. z o.o., Warsaw, Poland, Supplementary Material S1). The doses were chosen based on the previous study and observation for similar product^48^. The mix consisted of white mustard, calamus, turmeric, and common ivy. According to the producer’s declaration, this product contained herbal phytoncides, including phytoanticipins and phytoalexins, and the following nutrient content: 12.7% crude protein, 18.7% crude fiber, 7.5% ether extract, 7.5% crude ash, 0.54% lysine, 0.20% methionine, and less than 0.05% sodium. Birds had free access to feed and water throughout the trial. Composition of diets (Table 6) was the same as that previously published by Chodkowska et al.^48^.

In the first week of life, chicks were kept in electrically heated battery brooders with wire-mesh floor in groups of eight birds each and fed the appropriate experimental diet. The housing temperature was maintained at 32 ± 1 °C and light-dark cycle was set at 18 h of light and 6 h of dark. At eighth day of life birds were placed in an individual cages with wire-mesh floors and feeding the respective experimental diets was continued until the slaughter. During this period, chickens were kept at temperature of 32 °C, which was gradually decreased according to normal management practice, and at 18/6 h light-dark cycle. Body weight and feed intake were measured weekly. At 35 day of age chickens were sacrificed by decapitation and exsanguination. Blood was collected into heparinised tubes, centrifuged (3350*g*, 10 min, 4 °C), and plasma was stored at − 80 °C until further analyses. Pectoral muscle samples were taken, snap-frozen in liquid nitrogen and stored at − 80 °C. Blood and muscle tissue samples were collected from 10 birds per group.

**Table 6:** Ingredient and chemical composition of experimental diets for broiler chickens.

Ingredients (%)	Control diet			Diet with phytobiotics		
	Starter	Grower	Finisher	Starter	Grower	Finisher
Corn meal	32.44	32.07	31.80	32.44	32.07	31.80
Soybean meal	30.70	30.11	24.68	30.70	30.11	24.68
Wheat	30.00	30.00	35.00	30.00	30.00	35.00
Rapeseed oil	3.08	4.53	5.41	3.08	4.53	5.41
Premix with salinomycin	3.00	3.00	3.00	3.00	3.00	3.00
Monocalcium phosphate	0.41	0.24	0.11	0.41	0.24	0.11
Methionine	0.28	0.03	–	0.28	0.03	–
Lysine HCL	0.07	–	–	0.07	–	–
Calcium carbonate	0.02	0.02	–	0.02	0.02	–
Nutrients (%)—analyzed						
Dry matter	90.68	90.87	90.81	91.17	90.90	90.98
Crude protein	20.25	19.75	17.56	20.44	19.25	17.88
Crude ash	4.40	4.20	3.74	4.79	4.21	3.62
Crude fat	5.41	6.76	8.22	5.92	6.98	8.02
Crude fiber	2.54	2.67	2.76	2.48	2.62	2.69
Gross energy (MJ/kg)	17.6	18.2	18.6	18.2	18.9	18.8

### Chemical analyses of diets

Nutrient content in the experimental diets was analysed according to standard procedures^51^, while gross energy concentration was determined using a KL-12Mn adiabatic oxygen bomb calorimeter (Precyzja-BIT, Bydgoszcz, Poland).

### Total RNA isolation

Total RNA, including miRNA, was isolated from muscle tissue using miRNeasy Mini Kit (Qiagen, Hilden, Germany) according to the manufacturer`s protocol. The integrity of RNA was checked by electrophoresis on 2% agarose gel stained with ethidium bromide. The concentration and purity of RNA were measured spectrophotometrically using NanoDrop ND-1000 (Thermo-Scientific, Wilmington, DE, USA).

### Real-time qPCR

The expression of selected miRNA was analysed by the real-time quantitative PCR. The miRCURY LNA RT Kit (Qiagen, Hilden, Germany) was used for polyadenylation and reverse transcription into cDNA according to the manufacturer’s protocol. Gene expression analysis was performed using the miRCURY LNA SYBR Green PCR Kit (Qiagen) and specific primers (Table 7) designed using the GeneGlobe tool (http://geneglobe.qiagen.com), and miRNA sequences retrieved from the miRbase, the microRNA database (http://www.mirbase.org^52^). The reaction mix consisted of: 5 μl 2× miRCURY SYBR^®^ Green Master Mix, 1 μl PCR primer mix (Qiagen), 3 μl cDNA template (diluted 1:60), and 1 μl nuclease-free water. The PCR cycling conditions for miR-26a-5p, miR-30a-5p, miR-133a-5p, miR-142-5p, and miR-206 were as follows: initial activation at 95 °C for 2 min and 37 cycles of denaturation (95 °C, 10 s) and combined annealing/extension (56 °C, 60 s). For miR-99a-5p and miR-222 the number of cycles was reduced to 36, while for miR-181a-5p and U6 snRNA it was increased to 40 and 45, respectively. Reactions were performed on a MIC qPCR thermocycler (Bio Molecular Systems, Upper Coomera, QLD, Australia) and relative gene expression was calculated using the 2^−ΔΔCt^ method^53^ and U6 snRNA as a reference gene.

**Table 7:** The primers used in the qPCR assay of selected miRNA expression.

No.	Primer name	Target sequence	GeneGlobe ID
1	miR-26a-5p	gga-miR-26a-5p MIMAT0001118 UUCAAGUAAUCCAGGAUAGGC	YP02117348
2	miR-30a-5p	gga-miR-30a-5p MIMAT0001135 UGUAAACAUCCUCGACUGGAAG	YP00205695
3	miR-99a-5p	gga-miR-99a-5p MIMAT0001103 AACCCGUAGAUCCGAUCUUGUG	YP00204521
4	miR-133a-5p	gga-miR-133a-5p MIMAT0026509 AGCUGGUAAAAUGGAACCAAAUC	YP02106418
5	miR-142-5p	gga-miR-142-5p MIMAT0001193 CCCAUAAAGUAGAAAGCACUAC	YP02108747
6	miR-181a-5p	gga-miR-181a-5p MIMAT0001168 AACAUUCAACGCUGUCGGUGAGU	YP00206081
7	miR-206	gga-miR-206 MIMAT0001139 UGGAAUGUAAGGAAGUGUGUGG	YP00206073
8	miR-222	gga-miR-222 MIMAT0001107 CAGCUACAUCUGGCUACUGGGUCU	YCP1226287
9	U6 snRNA		YP00203907

### Target gene prediction and ontological analyses

MicroRNA target gene prediction was performed using the TargetScan Release 8.0 database (TargetScanHuman 8.0, David Bartel and Whitehead Institute Bioinformatics and Research Computing Group, USA). The analysis was performed for all phytobiotic-affected miRNAs. For each predicted target of individual miRNA, the sum of the context++ scores were automatically calculated^54^. Predicted targets of each miRNA family were automatically sorted by cumulative weighted context ++ score.

Ontological analyses revealing molecular functions, biological processes, and pathways of miRNA targets were performed in D.A.V.I.D. 6.7 (DAVID Functional Annotation Bioinformatics Microarray Analysis Bioinformatic Resources; Laboratory of Human Retrovirology and Immunoinfromatics, Frederic, MD, USA) using Fisher’s exact test with P ≤ 0.05. Detailed analysis of the role of phytobiotics-modulated miRNAs and target genes in various metabolic and signal pathways was performed using Panther 17.0 Classification System (http://www.pantherdb.org). Detailed analysis of the role of phytobiotic mixture on miRNAs and potential target genes in various metabolic and signal pathways was performed using Pathway Studio Web Software 12.5 (Elsevier, USA). Relationships between all differentially expressed miRNAs were visualized with Pathway Studio’s Build Pathway functionality which is based on the wave-propagation algorithm developed for the navigation through complex networks.

### Lipid peroxidation assay

The degree of lipid peroxidation in blood plasma and pectoral muscle was estimated spectrophotometrically based on the concentration of TBARS as described previously^48^. Before analysis, muscle samples were weighted to 0.2 g, homogenized in 0.8 ml of ice-cold 0.9% NaCl, and centrifuged (12,850*g*, 10 min, 4 °C), while blood plasma samples were assayed directly after 1 h-incubation of samples with 15% trichloroacetic acid and 0.37% thiobarbituric acid at 100 °C, followed by centrifugation (10,000*g*, 10 min). The absorbance was measured using a SpectraMax iD3 microplate reader (Molecular Devices, San Jose, CA, USA) at a wavelength of 532 nm. TBARS concentration was calculated from a standard curve for malonyldialdehyde.

### Analysis of prooxidant-antioxidant balance

The balance between oxidants and antioxidants was determined according to the method of Koliakos and Hamidi Alamdari^45^ based on simultaneous redox and enzymatic reactions. Muscle samples were prepared as described above. Briefly, 10 μl of blood plasma or muscle supernatant were placed into a 96-well plate. Then, 200 μl of the solution containing 3,3′,5,5′-tetramethylbenzidine, its cation, and horseradish peroxidase were added and incubated for 12 min at room temperature, in the dark. After the incubation, 100 µl of 2 M HCl solution was added to each well and incubated for 45 min. at room temperature, in the dark. Then, the absorbance was measured at 450 nm and 620 nm (reference wavelength) on a SpectraMax iD3 microplate reader (Molecular Devices, San Jose, CA, USA). The balance between oxidants and antioxidants was calculated from the standard curve prepared using 1 mM H_2_O_2_ solution as representative of oxidants and 6 mM uric acid solution as representative of antioxidants, and expressed in arbitrary units of Hamidi Alamadari and Koliakos (HK) per 1 ml of blood plasma or 1 g of tissue.

### Statistical analysis

Data are presented as mean ± standard error of the mean (SEM). Growth parameters and oxidative stress markers were analysed by one-way analysis of variance followed by Tukey’s HSD test. Data were checked for a compliance with the normal distribution by the Shapiro-Wilk test. All these analyses were performed using SPSS 11.5 software (SPSS Company, Chicago, IL, USA). The results of qPCR were analysed by a two-tailed Student’s *t*-test using the micPCR 2.10 programme (Bio Molecular Systems, Upper Coomera, QLD, Australia). P-values less than 0.05 were considered statistically significant.

### Institutional review board statement

The protocol of the study was approved by the animal welfare body of The Kielanowski Institute of Animal Physiology and Nutrition, Polish Academy of Sciences (Jabłonna, Poland), in accordance with the principles of the European Union and the Polish Law on Animal Protection. Reporting in the manuscript follows the recommendations in the ARRIVE guidelines.

## Conclusions

To our knowledge, this is the first study in which the effect of phytobiotic mixture, on selected miRNAs expression associated with muscle physiology was evaluated in broiler chickens. This preliminary study showed that the feed additive consisting of white mustard, calamus, turmeric, and common ivy may modulate the expression of miRNAs related to muscle cell proliferation, differentiation, hypertrophy, vitality etc. Moreover, it was observed that it also may influence the oxidative stress markers and miRNAs that are also involved in antioxidant activity. However, some of the results are not clear, which suggests the need for further research, including analyses of antioxidant enzymes activity. The results suggest that this unique phytobiotic composition may be a potential tool used in broiler chicken nutrition as a myoprotectant. Modulation of miRNAs by dietary factors seems to be very promising not only as potential therapeutic tool in animal disease but also may be helpful in reducing some of myopathies that affect the final meat quality and have an impact on the economic aspect of poultry production.

### Supplementary Information

Supplementary Information 1.

Supplementary Information 2.

Supplementary Table S3.

Supplementary Table S4.

## Data Availability

All data generated or analyzed during this study are included in this published article.
