# Supplementary material for: MicroRNA expression and oxidative stress markers in pectoral muscle of broiler chickens fed diets supplemented with phytobiotics composition
Source: Sci Rep. 2024 Feb 22;14:4413. doi: 10.1038/s41598-024-54915-y (PMC10884404; doi:10.1038/s41598-024-54915-y)

**Supplementary material**

**AdiMax®AP**

**Source of herbal phytoncides, including phytoanthicypines and phytoalexins, which have a beneficial effect on the digestive, hematopoietic and immune systems.**

**TARGETED SPECIES****:** laying hens (commercial layers and breeders), chickens for fattening, turkeys and waterfowl poultry.

**APPLICATION:** 150-300 g/t of complete feed.

**COMPOSITION** (feed materials): products from processing of spices and seasonings:  *Sinapis alba L.*; products from the processing of herbs: *Acorus calamus L., Hedera helix L.;* products from processing of spices and seasonings:  *Curcuma longa L.;* vegetable oil and fat: palm oil (hydrogenated).

**Additives** (in 1 kg):

**Flavouring compounds:** mixture of flavouring compounds /2b/: 10 000 mg

**Trace elements:** iron sulphate monohydrate /3b103/: 1,25% (Fe 4125 mg/kg), Zinc acetate dihydrate /3b601/: 1,25% (Zn 4000 mg/kg).

**Compulsory declaration:** crude protein: 12,70% (PN-A04018:1975+Az3:2002); crude fiber: 18,70%; crude fat: 7,50%; crude ash: 7,50%; lysine: 0,54%; methionine: 0,20%; sodium: <0,05%

Original Polish Version


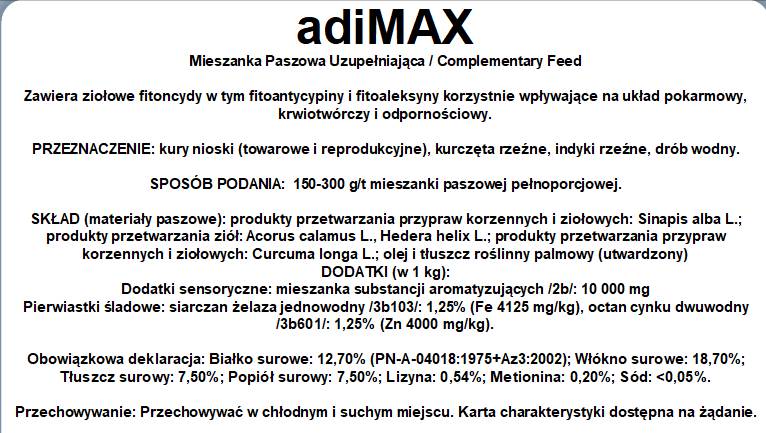

Supplement: Supplementary file 1 — Supplementary Information 1. [file 41598_2024_54915_MOESM1_ESM.docx]
