# Supplementary material for: MicroRNA expression and oxidative stress markers in pectoral muscle of broiler chickens fed diets supplemented with phytobiotics composition
Source: Sci Rep. 2024 Feb 22;14:4413. doi: 10.1038/s41598-024-54915-y (PMC10884404; doi:10.1038/s41598-024-54915-y)

**Effect of phytobiotic composition containing white mustard, calamus, turmeric and common ivy on miRNA expression in pectoral muscle, selected production parameters and oxidative stress markers in broiler chickens**

**Karolina A. Chodkowska, Marcin Barszcz, Anna Tuśnio**

Table of contents

1. Pathway studio summary list for miRNA; selected processes.

Table 1a. Protein regulators of cardiac hypertrophy. 1

Figure 1a. Protein regulators of cardiac hypertrophy-links between selected miRNAs and cardiac hypertrophy. 2

Table 1b. Protein regulators of myoblast proliferation. 2

Figure 1b. Protein regulators of myoblast proliferation-links between selected miRNAs and myoblast proliferation. 3

Table 1c. Protein regulators of skeletal muscle cell differentiation. 3

Figure 1c. Protein regulators of skeletal muscle cell differentiation -links between selected miRNAs and myoblast proliferation. 4

Table 1a. Protein regulators of cardiac hypertrophy.

**Jaccard similarity:** 3.24500E-3; **Overlap:** 6; **Percent Overlap:** 0; **Total # of Neighbors:** 1849; **p-value:** 3.13053E-7;

| **Name** | **Description** | **Object Type** | **InDegree** | **OutDegree** | **Total Connectivity** |
| --- | --- | --- | --- | --- | --- |
| **MIR206** | microRNA 206 | Protein | 0 | 1 | 1724 |
| **MIR99A** | microRNA 99a | Protein | 0 | 1 | 853 |
| **MIR222** | microRNA 222 | Protein | 0 | 1 | 1902 |
| **MIR142** | microRNA 142 | Protein | 0 | 1 | 1920 |
| **MIR26A1** | microRNA 26a-1 | Protein | 0 | 1 | 2212 |
| **cardiac hypertrophy** |  | Disease | 6 | 0 | 5124 |
| **MIR30A** | microRNA 30a | Protein | 0 | 1 | 1789 |


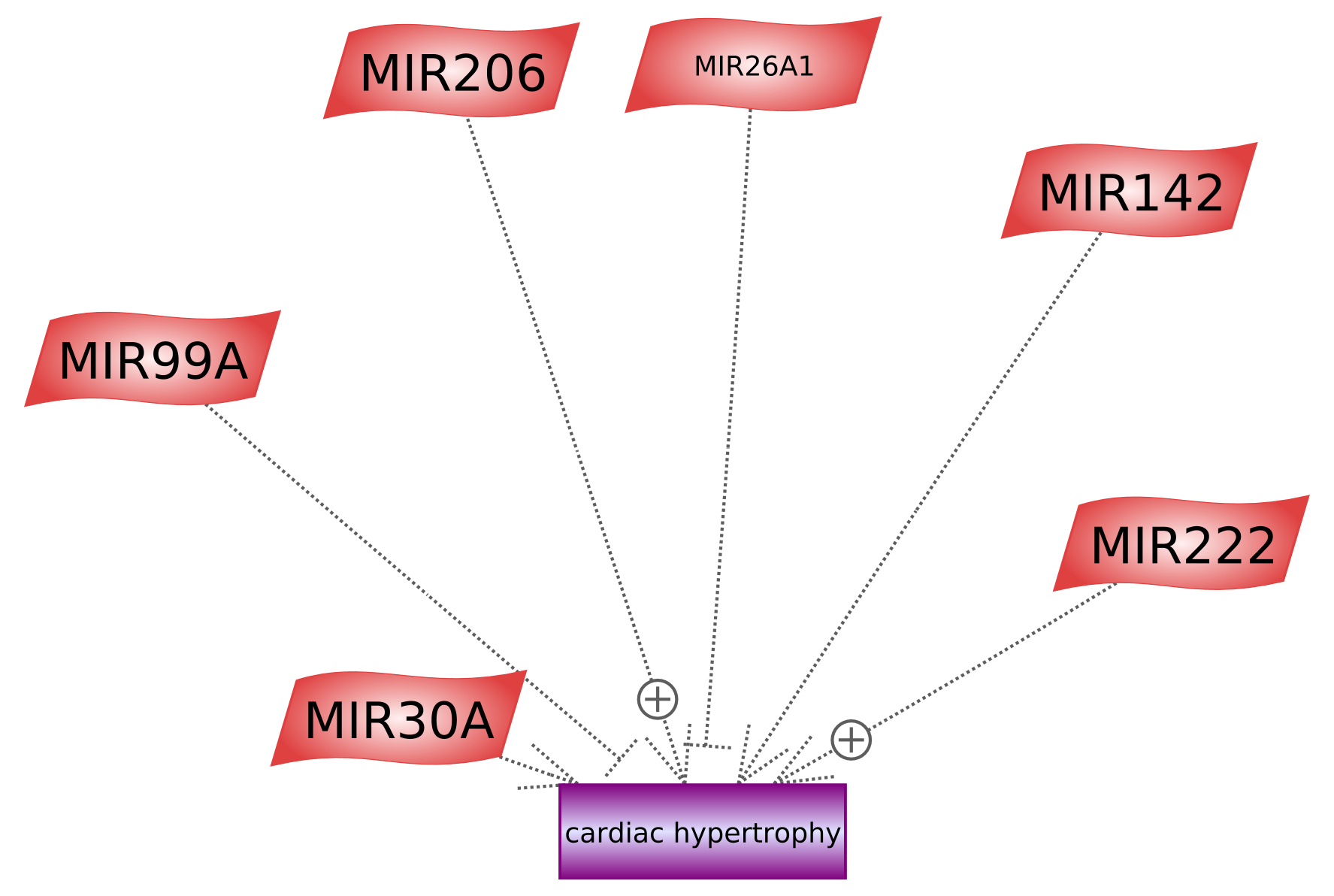


Figure 1a. Protein regulators of cardiac hypertrophy; links between selected miRNAs and cardiac hypertrophy. The shape of the arrows shows how the miRNAs affect hypertrophy.

Table 1a. Protein regulators of myoblast proliferation.

**Jaccard similarity:** 8.86918E-3; **Overlap:** 4; **Percent Overlap:** 0; **Total # of Neighbors:** 448; **p-value:** 2.81062E-5;

| **Name** | **Description** | **Object Type** | **InDegree** | **OutDegree** | **Total Connectivity** |
| --- | --- | --- | --- | --- | --- |
| **MIR206** | microRNA 206 | Protein | 0 | 2 | 1724 |
| **MIR222** | microRNA 222 | Protein | 0 | 1 | 1902 |
| **MIR142** | microRNA 142 | Protein | 0 | 1 | 1920 |
| **MIR26A1** | microRNA 26a-1 | Protein | 0 | 1 | 2212 |
| **myoblast proliferation** |  | Cell Process | 5 | 0 | 604 |


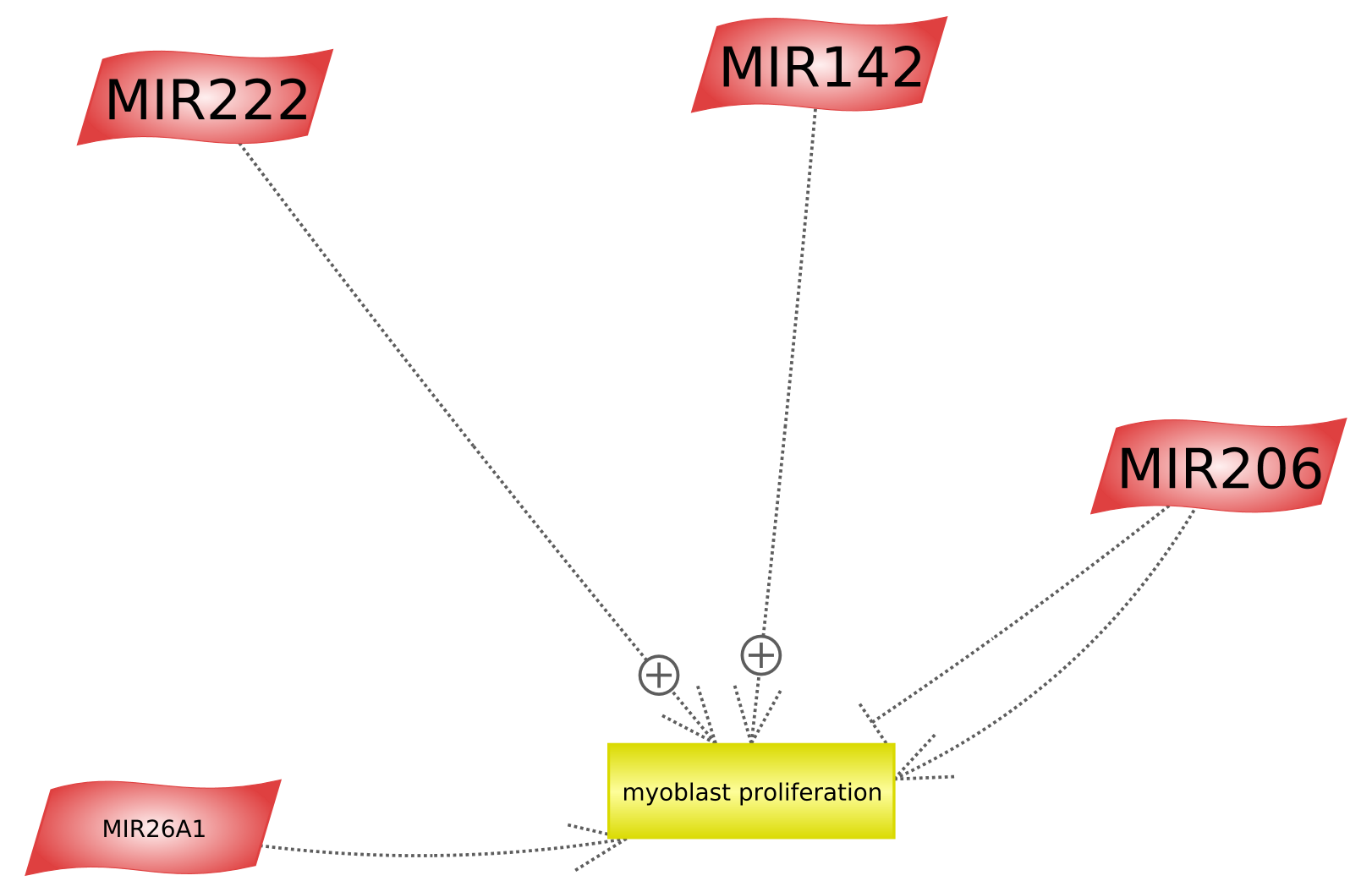


Figure 2a. Protein regulators of myoblast proliferation; links between selected miRNAs and cardiac hypertrophy. The shape of the arrows shows how the miRNAs affect myoblast proliferation.

Table 1c. Protein regulators of skeletal muscle cell differentiation.

**Jaccard similarity:** 1.65746E-2; **Overlap:** 3; **Percent Overlap:** 1; **Total # of Neighbors:** 177; **p-value:** 5.86237E-5;

| **Name** | **Description** | **Object Type** | **InDegree** | **OutDegree** | **Total Connectivity** |
| --- | --- | --- | --- | --- | --- |
| **MIR206** | microRNA 206 | Protein | 0 | 1 | 1724 |
| **skeletal muscle cell differentiation** |  | Cell Process | 3 | 0 | 209 |
| **MIR222** | microRNA 222 | Protein | 0 | 1 | 1902 |
| **MIR26A1** | microRNA 26a-1 | Protein | 0 | 1 | 2212 |

Figure 1c. Protein regulators of skeletal muscle cell differentiation -links between selected miRNAs and myoblast proliferation.


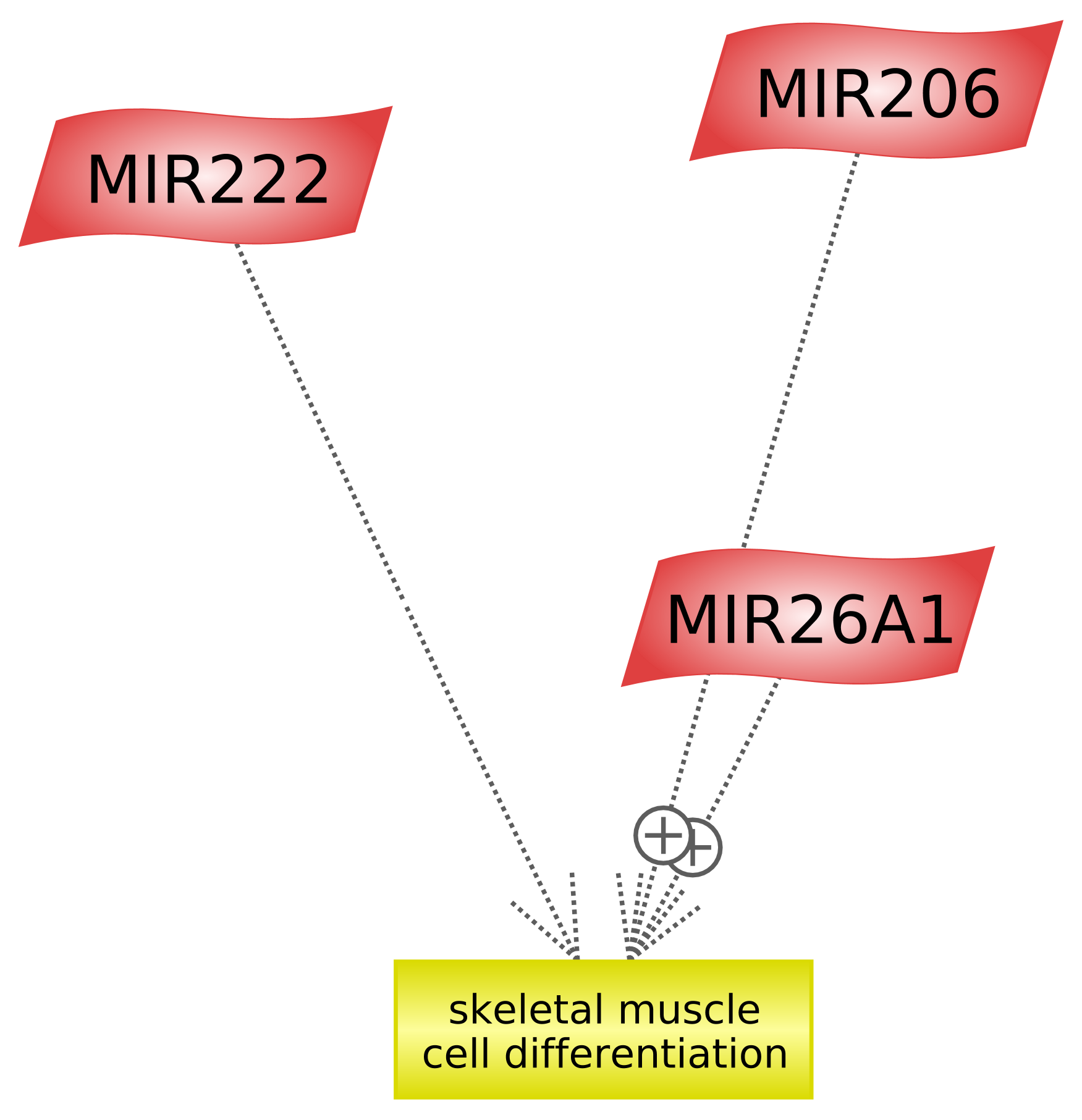

Supplement: Supplementary file 2 — Supplementary Information 2. [file 41598_2024_54915_MOESM2_ESM.docx]
